# PD207 Modelling Co-Financing Scenarios To Address The Financial Challenges Of Implementing A Colorectal Cancer Screening Program For Private Insurance Beneficiaries

**DOI:** 10.1017/S026646232400429X

**Published:** 2025-01-07

**Authors:** Carlos Muñoz-Montecinos, Camila Quirland, Felipe Maza, Carlos Barrientos, Catalina González-Browne

## Abstract

**Introduction:**

The World Health Organization (WHO) recommends population-based cancer screening programs for colorectal cancer (CRC). However, CRC screening is not available in Chile´s public health system. The Arturo Lopez Perez Foundation (FALP) is interested in implementing a CRC screening program for their insurance beneficiaries, despite some uncertainties regarding cost effectiveness and budget impact. Exploring co-financing scenarios could reveal feasible choices for program implementation.

**Methods:**

A Markov model was developed to assess the cost effectiveness of a biennial fecal immunochemical test (FIT) strategy for individuals aged 50 to 69 years from a private insurer perspective. Survival probabilities and average costs for each cancer stage at diagnosis were derived from our CRC patients. Benefits were measured as life-years gained. The budget impact was calculated using the expected population of beneficiaries for 20 years. Cost-effectiveness results were reported using the incremental cost-effectiveness ratio (ICER), with a referential threshold of one to three times the gross domestic product per capita (USD16,000 to USD48,000). Shared funding scenarios with national public insurers and out-of-pocket payments were simulated.

**Results:**

CRC screening was cost effective in the base case and shared funding scenarios. Indeed, it showed some benefits and savings in the latter scenario. On the other hand, budget impact values were unaffordable for the base case scenario, revealing that implementing the program would be difficult even with the promising ICER. However, in shared funding scenarios, the budget impact decreased and even showed savings after five years due to the decrease in cancer treatment costs.

**Conclusions:**

CRC screening with a biennial FIT is a cost-effective strategy for FALP beneficiaries, but the budget impact is substantial and poses challenges for its implementation. The use of shared funding strategies could help alleviate the budget impact, making the implementation of a screening program more feasible.

